# Health Issues of Primary School Students Residing in Proximity of an Oil Terminal with Environmental Exposure to Volatile Organic Compounds

**DOI:** 10.1155/2016/4574138

**Published:** 2016-07-03

**Authors:** Massimo Cipolla, Marco Bruzzone, Emanuele Stagnaro, Marcello Ceppi, Alberto Izzotti, Claudio Culotta, Maria Teresa Piccardo

**Affiliations:** ^1^Mutagenesis Unit, IRCCS AOU San Martino-IST, Istituto Nazionale Ricerca sul Cancro, 16132 Genoa, Italy; ^2^Clinical Epidemiology Unit, IRCCS AOU San Martino-IST, Istituto Nazionale Ricerca sul Cancro, 16132 Genoa, Italy; ^3^Department of Health Sciences, University of Genoa, 16132 Genoa, Italy; ^4^Epidemiology Unit, Azienda Sanitaria 3 Genovese, 16149 Genoa, Italy

## Abstract

Residential proximity to industrial sites has been associated with adverse effects on human health. Children are more susceptible to airborne environmental exposure because their immune and respiratory systems are still developing. This study aimed to investigate whether living close to an oil terminal in Genoa where there is higher VOCs exposure is associated with an increased rate of school absenteeism because of disease in primary school children. Five schools were chosen for the recruitment of children and students residing in the industrial site (A) were compared to those living in residential sites (B). Sixty-six of the 407 students involved in the project were also selected for VOC monitoring. Source apportionment was carried out by comparing profiles of VOCs; principal component analysis was performed to study the correlation between profiles, and Kriging interpolation model was used to extend profiles to all participants. The concentration means of total VOCs were significantly higher in the industrial areas compared to controls. Adjusting for potential confounders, children who lived in area A had a significantly higher risk of being absent from school due to sore throat, cough, and cold compared to controls.* o*-Xylene, which is dispersed during the industrial activity, showed clear evidence of a significant association with respiratory symptoms.

## 1. Introduction

Different human activities emit volatile organic compounds in urban and industrial atmospheres; in particular, VOCs are emitted by traffic via vehicle exhausts and fuel evaporation, residential heating, industry energy production, solvent usage in industrial processes and solvent industries, and petroleum storage and distribution [1]. Accidental leaks as well as regulated emissions can contribute to the ambient concentrations of VOCs.

Anthropogenic and biogenic VOCs react to form secondary organic aerosols contributing to ambient fine particulate matter (PM_2.5_) [2] and these also play important roles in the photochemical reactions that form the ozone [3, 4].

The Environmental Protection Agency (EPA) has listed some VOCs as “hazardous air pollutants” (https://www3.epa.gov/ttn/atw/188polls.html), and several genotoxic effects of subchronic low-dose VOCs mixture have been recognized [5]. Some VOCs, such as benzene, toluene, ethylbenzene, and xylene (BTEX), have endocrine disrupting activity [6] and their exposure in humans can induce genetic and epigenetic change, altering microRNAs expression and DNA methylation [7, 8].

Exposure to VOCs individually and mixtures can cause a wide range of adverse effects on human health, including respiratory irritation, asthma exacerbation, and allergy [9].

VOC emissions from oil terminals and storage tanks and during loading/unloading operations into the oil tanker are of great concern because not only workers but also nonoccupationally exposed populations living nearby may be exposed [10].

Special attention has been focused on the child population. Children are more susceptible to airborne environmental exposures because their lung function is not completely developed [11]. The association between asthma symptoms in children and petrochemical pollution has been demonstrated in several studies [12, 13]. Other evidences show that children living in proximity to a petroleum refinery area, when compared with the control zone, have a prevalence of respiratory hospitalizations and nocturnal cough [14] and also a decrease in lung function and increased markers of bronchial inflammation and oxidative DNA damage [15]. Asthma and respiratory disorders in children are important health concerns in relation to quality of life and also in terms of health care costs and absences from school [16].

The aim of this study was to evaluate whether living close to an oil terminal with consequent exposure to VOCs was associated with increased rates of school absenteeism due to diseases. The analyses of the adverse effects of VOCs on health have been conducted by using outdoor VOCs, as utilized in previous studies [12–17]. We also assessed the possible correlation between individual VOC profiles and absences for health reasons.

## 2. Materials and Methods

### 2.1. Study Area

Genoa is the most important city of the Ligurian region and, with a population of 594,774 inhabitants (2014), it is the sixth largest city in Italy. The coastline is spread out over 42 kilometres with the western area more industrial and the eastern area more residential. The oil terminal of the Genoa harbour, situated in the western part of the city, covers an area of 123,000 square metres, and it consists of one dock that is used for the loading/unloading of chemical products and four landing stages used to discharge crude oil and to load/unload manufactured products (approximately 15 mtons/year). A network of pipelines connects the oil terminal to several refineries in northern Italy and Switzerland. Two storage spaces specialized for the receipt, storage, and forwarding of chemicals and petrochemicals by road, railway, and sea are directly connected to the oil terminal. VOCs are the main handled products.

**Figure 1:**
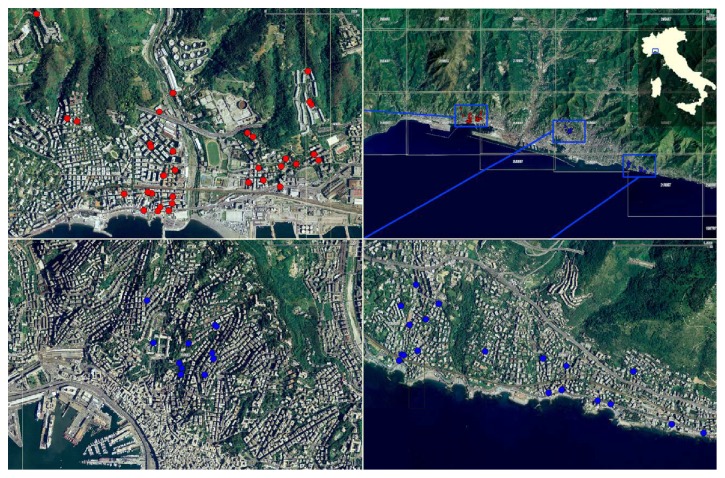
Map of the studied areas and VOC sampling points. The four images from the right in a counterclockwise direction are (1) the study area; (2) industrial area A (red dots); (3) the residential downtown section (control area B) (blue dots); (4) the eastern section of the city (control area B) (blue dots).

The petrochemical complex is located in an urban context where approximately 25,000 people live. Houses and petrochemical plant coexist on the territory. Furthermore, this area (industrial area A) is characterized by other potential VOC sources, that is, a ship dockyard, a motorway, and urban traffic. To investigate the health effects of exposure to VOCs on people in the area, a control section of the downtown and a section of the eastern part of the city were chosen that were free from industrial activity and far from the fallout of Genoa harbour (control area B). The downtown section is about 8 km far from the industrial area A and the eastern section of the city is 8 km far from the downtown. The studied areas are represented in Figure 1.

### 2.2. Study Participants

Five primary schools were chosen for the recruitment of children to be participants in the study. The study was performed in 2006 for two months, that is, from October 16 to December 16.

Schools were chosen as surrogates for residential places and divided in two areas with different exposures. Two schools were located in the “industrial area A” with a small distance from the petrochemical activity, and three schools were located in the “control area B,” outside of the industrial activity, and were distant from the oil terminal of Genoa harbour. The motivation to investigate primary school students was due to the scholastic population living in the same area where the school was located.

A total of 644 students (aged 6–10 years) were initially selected for the present study: 235 attended the schools in the industrial area and 409 lived in the control area. The educational authorities and teachers of the five schools gave their consent and cooperated with the study. They arranged a meeting with the parents of potential participants to explain the nature of the study and to ask them for written informed consent to allow their children to participate in the study.

A total of 74 of 644 students involved in the project were also selected for two VOC monitoring campaigns; of these, 39 belonged to the “industrial area A” and 35 belonged to the “control area B.” The characteristics required were being representative of the studied areas and residing on the 3rd floor or higher to observe the distribution of VOC pollution not in close contact with traffic sources. These children were also trained on the passive sampler functions and its activation. Participation in the study was voluntary.

### 2.3. Questionnaires

Detailed information was obtained by a questionnaire based on instruments used in Italian investigations [18, 19], in the framework of the international study of asthma and allergies in childhood (ISAAC Project) [20]. The questionnaire reported in Supplementary Material available online at http://dx.doi.org/10.1155/2016/4574138 (1S) included residential history, house information (area, floor level, heating and ventilation type, and presence of mould), family (composition, educational level of parents, and smoking habits), use of candles, and/or incense and the presence of pets.

A second set of questions collected the medical history of the child. It concerned chronic respiratory diseases, such as asthma, and recurrent respiratory infections, as well as symptoms such as unusual fatigue and sleeplessness and prevalence of allergic diseases. Finally, the questionnaire included a register to record the child's school absences with a medical explanation (cough, cold, and others) for each day throughout the study period.

School teachers distributed the questionnaires and registers on October 16, and then they collected them during the week of December 18 after the questionnaires were completed by the parents.

### 2.4. VOC Sampling

PerkinElmer (Perkin Elmer Italia S.p.A.) stainless steel sample tubes prepacked with Chromosorb 106 (60/80 mesh) and previously conditioned as discussed elsewhere [21, 22], were distributed to the children for passive VOC monitoring. They exposed the samplers outside of their home following the written instructions supplied with the questionnaire. Two samplings were performed during the following periods, that is, from October 23 to November 6, 2006, and from December 3 to December 17, 2006.

### 2.5. VOC Analyses

VOC samples were analysed using a Clarus 500 gas chromatograph with a flame ionization detector (PerkinElmer) and a PerkinElmer PE-5 capillary column (30 m × 0.25 mm × 0.25 mm) [21, 22].

All peaks with retention times (RT) between 12 min and 55 min that were present in both monitoring campaigns were examined (total VOCs); those exceeding a height set at 5 percent of the toluene peak of each sample were taken for profile evaluation; their concentrations, calculated using the toluene calibration [22], were expressed as the toluene equivalent. An identification code was assigned to each peak, formed by a capital letter corresponding to the initial letter of the reference peak and by an ordinal number corresponding to the position of the peak compared to the reference compound. Benzene, toluene, and* p*-xylene were reference peaks that represented B0, T0, and X0, respectively.

In order to calculate BTEX concentration, a calibration curve was obtained by injecting each sampling tube with 1 *μ*L of a standard solution containing a known amount (from 0.2 to 100 ng mL^−1^) of pure BTEX (from 99.5% to 99.9%) dissolved in methanol (Toxic Organic Mix 1A; Supelco). The mass of analyte was determined from the gas chromatographic area and corrected by subtracting the average blank area according to the calibration curve [22]. Field blanks and triplicate samples were used to verify the sampling methodology. Field blanks were analysed to determine the limit of detection (LOD) and to set the zero concentration for each analytical run. The LOD for each BTEX, calculated as the average value of the field blanks plus 3 times the standard deviation, was 0.1 *μ*g/m^3^.

To select samples of predominantly industrial or urban origin, source apportionment was carried out by comparing the profiles of VOCs. VOC profiles were calculated as the ratio of the concentration of 37 selected peaks to that of ethylbenzene measured in the same sample. Ethylbenzene was chosen because it represents the traffic emissions and is not handled in the oil port.

### 2.6. Statistical Methods

The mean and standard deviation (SD) were computed for VOC concentrations of A and B areas, and* t*-tests were performed to compare the two zones.

A Chi-squared test (*χ* ^2^) was applied to describe and evaluate the heterogeneity of the distribution for both dwelling and family characteristics [23].

Principal component analysis (PCA) [24] was performed to study the correlation between profiles; graphics were produced for both profiles and spaces to explore whether individuals and profiles showed a similar pattern. If so, the profiles along with the reference peaks for B0, T0, X0, and X14 were taken into account for regression analysis as predictors of the number of days of absence caused by the different symptoms considered.

To extend the VOC profiles derived from chemical monitoring of all 407 participant students, the Kriging interpolation model was used [25]. This model uses the surrounding measured values to obtain a predicted value for an unmeasured location. Kriging is founded on regionalized variable theory, which supposes that the spatial variation in the data being modelled is homogeneous across the area in question.

Regression models were fitted to the data assuming that the number of days of absence follows a negative binomial distribution (NB), which is the statistical model more suitable for counts [26].

The NB model estimates the ratio between the average absence rate observed in the levels of each covariate included in the model compared to the reference level, that is, the mean ratio (MR). For each symptom, an “*ad hoc*” regression model was built, performing a backward stepwise procedure to identify covariates that were more predictive. A log-likelihood ratio test (LRT) was performed to test the contribution of each covariate to the statistical model, and 95 percent confidence intervals (95% CI) were computed.

## 3. Results 

A total of 553 children among the 644 originally identified agreed to participate in the study. Completed questionnaires were obtained from 407 children (response rate 74%), of which 152 attended the schools of the industrial area A and 255 attended those of the control area B.

Among 74 children selected for VOC measurements, 66 successfully completed both monitoring campaigns (response rate 89%), with 35 belonging to the industrial area and 31 to the control area. Compliance was similar in the two areas.

Thirty-seven peaks with retention times between 12 min and 55 min were taken into account in this study. The concentration mean* toluene equivalent* (*μ*g/m^3^) of total VOCs (VOCtot) and the sum of 37 VOCs (∑37 VOC) measured by 66 students of five primary schools during the two monitoring campaigns in the industrial and control areas are presented in the Supplementary Material (Supplemental Table  1S). For both sampling areas, the mean value, standard deviation, and minimum and maximum values were also calculated. A wide variation in VOC levels was highlighted between sampling sites of both areas, as shown by the minimum and maximum levels in the industrial area and in the control area. The concentration means of the total VOC were higher in area A (114.4 ± 61.3 *μ*g/m^3^) compared to control area B (80.7 ± 33.4 *μ*g/m^3^), and these differences resulted in a statistically significant* t*-test result (*p* = 0.004). Additionally, ∑37 VOC concentration means were higher in area A (52.9 ± 27.2 in area A and 40.9 ± 17.7 *μ*g/m^3^ in area B), and these differences were also statistically significant (*p* = 0.020).

The concentration means of BTEX were not different in the industrial and control areas, except for* o*-xylene (X14), which was not correlated with its isomers* m*-xylene and* p*-xylene, as shown in Figure 4, while a good correlation was present in the control area. In the industrial area* o*-xylene was also well correlated with the other VOCs ∑(T5 + T24 + X5 + X22)  (*R* ^2^ = 0.787) and T5 correlated with T25 (*R* ^2^ = 0.829). These correlations were not present in area B.

The variables in the questionnaire were analysed, and the frequencies in the levels of the more notable features for each zone are reported in Table 1. Only age and floor of residence were not independent in the two zones, as is shown by *χ* ^2^ test results.

Table 2 shows peaks of 37 VOCs present in both areas, whose concentration means were significantly higher in proximity of the oil terminal area.

**Figure 2:**
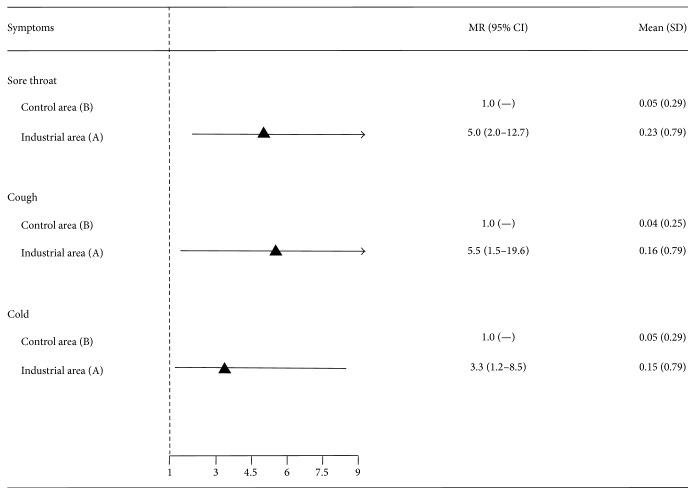
Days absent due to respiratory symptoms: estimation of mean ratios (MRs) by area of residence with a negative binomial regression model.

The comparison between zones A and B concerning days of absence for specific symptoms performed by the NB regression model is reported in Figure 2. Adjusting for potential confounders, children living in zone A had a significantly higher risk of school absenteeism due to sore throat (MR = 5.0; 95% CI 2.0–12.7), cough (MR = 5.5; 95% CI 1.5–19.6), and cold (MR = 3.3; 95% CI 1.2–8.5) compared to controls.

**Figure 3:**
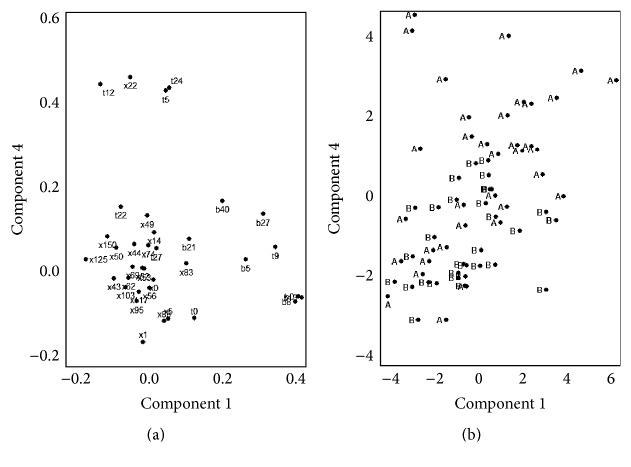
Principal component analysis: plot of components 1 and 4 in the space of profiles (a) and in the space of individuals labelled by zone of residence (b).

**Figure 4:**
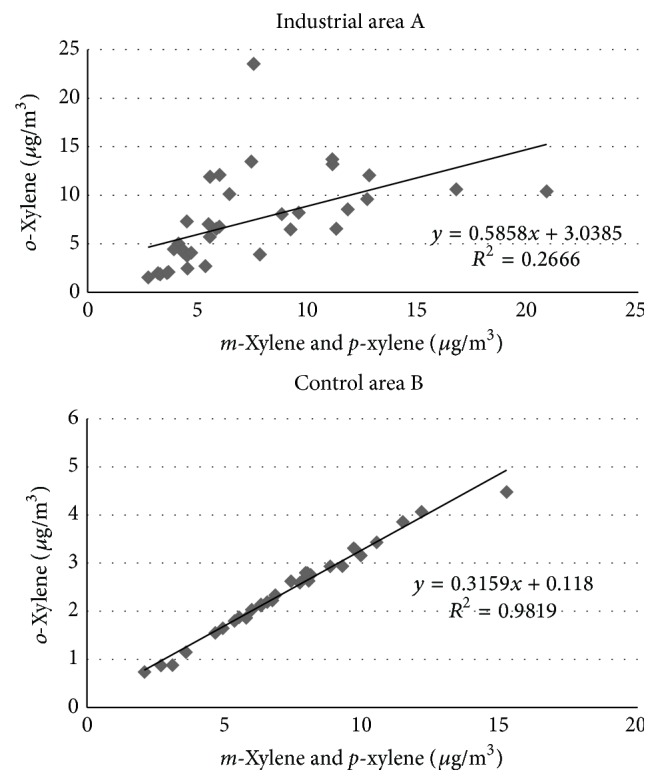
VOC correlations.

Figure 3 shows the profile distribution as a result of PCA. Ten components were considered, which explained approximately 80% of the total variance. For components 1 and 4, T5, T12, T24, and X22 profiles presented a similar pattern to the observations sampled in zone A.

Therefore, the ratios of these peaks (T5, T12, T25, and X22) with ethylbenzene as well as benzene, toluene,* p*-xylene,* m*-xylene, and* o*-xylene (BTX) were included one by one in the regression models as supposed predictors of the number of absence days. BTX were also considered because they are traffic-related VOCs of public health importance, included in well-established control plans (EU Directive 2008/50/EC).

The correlations of these peaks, divided by tertiles of their frequency distribution, with days of absence for sore throat, cough, and cold were the only symptoms that showed significant results and are reported in Table 3. Sore throat was correlated with the following ethylbenzene ratios: B0/E, T5/E, T24/E, X1/E, X13/E, and X22/E; cough was correlated with T0/E, T24/E, X14/E, and X22/E; cold was correlated with T5/E, T24/E, X0/E, and X14/E. High MRs were also observed for X14/E, corresponding to the* o*-xylene/ethylbenzene ratio, which was a concern for all three symptoms. We also tested the occurrence of trends among tertiles of the profiles, and it was noteworthy that X14/E presented a linear increase in all the symptoms considered. Trends were also evident for T5/E, T24/E, and X22/E concerning sore throat.

## 4. Discussion

According to our results, during the study period, the industrial area was more polluted than the control area (Table  1S), and there was a higher frequency of absences for respiratory syndromes in zone A (Figure 2). These results are consistent with what was reported by Ware et al. [27] for a 10 *μ*g change of petroleum-related VOCs and an increased cumulative incidence of lower respiratory symptoms (OR 1.05; 95% CI 1.02–1.07).

For urban air and traffic air samples,* m*-xylene,* p*-xylene, and* o*-xylene are always correlated [28]. The lack of correlation between xylenes isomers in the industrial area A and the presence of other compounds* o*-xylene correlated suggest a common source of VOCs pollution.

This assertion is supported by measurements of the air pollution control, according to D.Lgs. n. 267/2000, performed from 2002–2005 near the oil terminal area by the Ligurian Environmental Protection Agency (data by Provincia di Genova) [29]. This study highlighted a continuous reduction of carbon monoxide (CO) levels that were not correlated with the increase of nonmethane hydrocarbons (NMHCs). This was in disagreement with studies of motor vehicle emissions that showed that NMHCs decreased at a similar rate as CO [30]. Moreover, according to data by “Provincia di Genova” [29], weekly sampling campaigns of BTEX performed by diffusive passive samplers highlighted that xylene concentrations did not show seasonal correlations, which was a clear sign that xylene emissions could not be attributed to traffic pollution but were emitted from a different source. In fact, air quality monitoring studies carried out in urban cities indicated that significant seasonal variability of BTEX was present [31, 32], and the lack of seasonality in BTEX could be attributed to the local VOC source distribution [32].

Regarding the VOC profile analysis, we found a clear significant trend between* o*-xylene exposure and school absences for all three symptoms (i.e., sore throat, cough, and cold). On the other hand, as reported by a previous study [33], the inhalation of xylene vapours can cause irritation of the nose and throat, causing cough and/or difficulty breathing. These data are also in agreement with results reported in some occupational studies that showed increases in lower respiratory inflammations due to exposure to mixtures of xylenes [34, 35].

Experimental studies have shown that exposure to a single VOC could induce an inflammatory response as well as a variety of irritating symptoms in the airway [35–38].

Additionally, for the other VOCs and* o*-xylene correlations (T5, T24, X5, and X22), we observed higher risks for illness absences, confirming the same possible source of pollution in the industrial area (Table 3). The traffic-related VOCs, such as toluene and, above all, benzene, the most known and investigated compound due to its carcinogenic effects [39], seemed to be less associated with school absences for health reasons. Also meta- and para-xylene did not have a particular effect on school absences compared to ortho-xylene because of being in smaller quantities.

Our data could be related to short-term exposure to VOCs, as discussed in a previous study [14], where a higher prevalence of respiratory hospitalizations and nocturnal cough was found in children and adolescents living near petrochemical sites.

Short-term exposure to VOCs, in particular toluene, ethylbenzene, and xylene has been associated by Hong et al. [7] with change on genes expression related to respiratory systems. Additionally, data from different investigations supported the hypothesis that there was an associative effect of VOC exposure and worse respiratory health, such as asthma, wheezing, dyspnoea, nocturnal cough, rhinitis, and lower lung function, in children living near a petrochemical complex compared to those in urban and residential areas [12, 15]. On the other hand, children were more susceptible to the negative effects of air pollution than adults due to their developing immune and respiratory systems and because they inhaled more air relative to their body size [40, 41].

Several investigations have focused attention on children's exposure to VOCs, as largely influenced by the indoor air quality at school and at home [42–44]. The relationships between indoor pollution and short- and long-term health problems in children were highlighted [45].

The indoor concentrations of VOCs are generally higher than those of outdoor air [46, 47]; however, the indoor air concentration reflects the external ambient air. The highest levels of VOCs, as measured by Jia et al. [48], both outside and inside residences of industrial areas compared to those of suburban and urban areas, confirmed an indoor-outdoor relation of VOC levels. This evidence was also supported by the indoor seasonal effect found only in the industrial sites, due to a change in ambient levels [49].

In our study, the lack of indoor VOC measures was balanced by information derived from the questionnaire for the indoor pollution data by including analysis of the smoking habits of the parents, the smoke from candles and incense, heating types, the presence of mould and dampness, and the habit to refresh the indoor air by opening a window, which was used as a surrogate in the model. The results for these potential confounders were in partial agreement with previous studies that found weak associations. Moreover, the analysis was adjusted for all these variables in addition to gender and age.

The strength of our study was recording both the absences day per day with the health information to minimize any recall bias and measurements of the environmental pollution VOCs during the same period.

The main limitation of our study was the small size of our sample and the relatively brief period of observation, that is, only one season (autumn). The levels of pollutants could differ in other periods due to seasonal effects, and there could be changes in the life habits of children, such as spending more or less time outdoors.

## 5. Conclusions 

Although there were some limitations, the results of our study are in good agreement with those reported in previous investigations; that is, children living in petrochemical industrial areas show an association between exposure to VOCs and increases in lower respiratory diseases. Our findings supported a relationship between higher* o*-xylene levels and sore throats, coughs, and colds, while benzene, toluene, and* m*-xylene and* p*-xylene exposures seemed to be only partially associated with absences for health reasons.

To fully understand the impact of VOC pollution and the potential synergies among these compounds and the respiratory health of children, a wider study is needed. A future study should be carried out in at least two different seasonal periods where qualitative and quantitative determinations of a broader spectrum of VOCs could be performed both inside and outside buildings.

## Supplementary Material

(Table 1S) Questionnaire for epidemiologic investigation in primary schools of Genoa (Italy).

## Figures and Tables

**Table 1:** Distribution of children by gender, age, smoking habits, and house information.

Characteristic	Industrial area (A)		Control area (B)		*χ* ^2^	*p* value
	*N*	%	*N*	%		
Gender					1.17	0.279
(i) Male	83	55.0	126	49.4		
(ii) Female	69	45.0	129	50.6		
Age					**11.72**	**0.020**
(i) 6	3	2.0	10	3.9		
(ii) 7	15	9.9	56	22.0		
(iii) 8	38	25.0	58	22.7		
(iv) 9	48	31.6	67	26.3		
(v) 10	48	31.6	64	25.1		
Smoke in family					0.67	0.412
(i) No	117	77.0	205	80.4		
(ii) Yes	35	23.0	50	19.6		
Humidity/mould					3.25	0.071
(i) No	125	82.8	190	75.1		
(ii) Yes	26	17.2	63	24.9		
Allergies					0.03	0.857
(i) No	128	84.2	213	83.5		
(ii) Yes	24	15.8	42	16.5		
Years of residence					0.01	0.936
(i) ≤2	15	10.1	26	10.3		
(ii) >2	134	89.9	226	89.7		
Floor of residence					**6.53**	**0.011**
(i) <3	65	43.0	140	56.2		
(ii) ≥3	86	57.0	109	43.8		

Total	152		255			

For some characteristics, *N* values do not add up to the total because of missing values.

**Table 2:** Concentration means and standard deviations (*μ*g/m^3^) of peaks measured in the industrial and control areas that were significantly different according to *t*-test.

Peak	Industrial area (A)	Control area (B)	*p*
	*µ*g/m^3^	*µ*g/m^3^	
B5	0.4 ± 0.5	0.1 ± 0.5	0.044
B27	1.0 ± 0.6	0.7 ± 0.4	0.003
T5	0.3 ± 0.2	0.2 ± 0.1	0.004
T24	0.6 ± 0.5	0.2 ± 0.2	<0.001
X5	1.0 ± 1.0	0.1 ± 0.2	<0.001
X14	7.3 ± 4.7	2.4 ± 0.9	<0.001
X22	0.6 ± 0.4	0.3 ± 0.5	0.005
X44	0.8 ± 0.7	0.1 ± 0.2	<0.001
X125	0.8 ± 1.2	0.1 ± 0.1	0.001

**Table 3:** Days absent due to respiratory syndromes: estimation of mean ratios (MR) by tertiles of selected profiles through the negative binomial regression model.

	Sore throat			Cough			Cold		
	Mean (SD)	MR (95% CI)	*p* value trend	Mean (SD)	MR (95% CI)	*p* value trend	Mean (SD)	MR (95% CI)	*p* value trend
B0/E									
<0.85	0.04 (0.27)	Ref.	0.090						
0.85–1.01	0.16 (0.64)	5.0 (1.3–18.9)							
>1.01	0.15 (0.61)	3.3 (0.9–12.2)							
T0/E									
<5.94				0.05 (0.31)	Ref.	0.821			
5.94–7.12				0.20 (0.95)	4.8 (1.1–21.7)				
>7.12				0.02 (0.13)	0.4 (0.1–2.6)				
T5/E									
<0.08	0.04 (0.27)	Ref.	**0.016**				0.06 (0.43)	Ref.	0.087
0.08–0.14	0.11 (0.49)	3.1 (0.8–11.8)					0.04 (0.31)	0.8 (0.2–3.4)	
>0.14	0.19 (0.74)	4.9 (1.4–17.6)					0.18 (0.60)	5.4 (1.3–21.8)	
T12/E									
<0.06									
0.06–0.11									
>0.11									
T24/E									
<0.07	0.04 (0.27)	Ref.	**0.002**	0.05 (0.31)	Ref.	0.056	0.06 (0.43)	Ref.	0.092
0.07–0.18	0.06 (0.30)	1.6 (0.4–6.2)		0.02 (0.19)	0.3 (0.0–2.2)		0.02 (0.13)	0.3 (0.1–1.5)	
>0.18	0.24 (0.82)	6.5 (1.9–21.8)		0.20 (0.94)	5.7 (1.2–26.9)		0.20 (0.65)	3.1 (1.1–9.2)	
X0/E									
<2.76							0.06 (0.43)	Ref.	0.114
2.76–2.79							0.05 (0.32)	1.0 (0.2–4.2)	
>2.79							0.17 (0.59)	4.9 (1.2–20.0)	
X1/E									
<1.24	0.04 (0.27)	Ref.	0.269						
1.24–1.26	0.21 (0.75)	6.4 (1.8–23.0)							
>1.26	0.09 (0.48)	2.1 (0.5–8.0)							
X14/E									
<1.35	0.04 (0.26)	Ref.	**0.003**	0.01 (0.12)	Ref.	**0.005**	0.01 (0.12)	Ref.	**0.001**
1.35–2.95	0.06 (0.32)	1.8 (0.4–7.7)		0.07 (0.38)	4.5 (0.6–33.3)		0.08 (0.50)	5.8 (1.0–32.8)	
>2.95	0.22 (0.78)	6.1 (1.7–21.2)		0.18 (0.88)	15.9 (2.2–115.1)		0.18 (0.62)	13.3 (2.5–69.6)	
X22/E									
<0.12	0.04 (0.27)	Ref.	**0.002**	0.05 (0.31)	Ref.	0.055			
0.12–0.15	0.06 (0.30)	1.6 (0.4–6.2)		0.03 (0.21)	0.5 (0.1–3.0)				
>0.15	0.24 (0.82)	6.5 (1.9–21.9)		0.19 (0.94)	5.1 (1.1–24.2)				

Bold *p* values indicate statistically significant.
