# Supplementary material for: Health Issues of Primary School Students Residing in Proximity of an Oil Terminal with Environmental Exposure to Volatile Organic Compounds
Source: Biomed Res Int. 2016 Jul 3;2016:4574138. doi: 10.1155/2016/4574138 (PMC4947509; doi:10.1155/2016/4574138)
Supplement: Supplementary file 1 — (Table 1S) Questionnaire for epidemiologic investigation in primary schools of Genoa (Italy). [file 4574138.f1.pdf]

Health issues of primary school students residing in proximity of an oil terminal with environmental exposure to volatile organic compounds

Massimo Cipolla[a], Marco Bruzzone[b], Emanuele Stagnaro[b], Marcello Ceppi[b], Alberto Izzotti[ac], Claudio Culotta[d], Maria Teresa Piccardo[a]*

[a] Mutagenesis Unit, IRCCS AOU San Martino – IST, Istituto Nazionale Ricerca sul Cancro, Genoa, Italy

[b] Clinical Epidemiology Unit, IRCCS AOU San Martino – IST, Istituto Nazionale Ricerca sul Cancro, Genoa, Italy

[c] Department of Health Sciences, University of Genoa, Genoa, Italy

[d] Epidemiology Unit, Azienda Sanitaria **3** Genovese, Genoa, Italy.

*Corresponding author: Maria Teresa Piccardo

e-mail address: mariateresa.piccardo@hsanmartino.it

mailing address: U.O.C. Mutagenesi, IRCCS AOU San Martino – IST, Istituto Nazionale Ricerca sul Cancro, Largo R Benzi n 10, 16132 Genoa, Italy

Table 1S

Concentration means (µg/m^3) of VOCtot and of Σ37 VOC measured during two monitoring campaigns performed in the industrial and control areas

Industrial area (A)			Control area (B)		
Sample	**VOCtot**	**Σ37 VOC**	***Sample***	**VOCtot**	**Σ37 VOC**
1	123.3	49.3	36	106.8	52.9
2	220.7	116.4	37	109.9	59.7
3	88.8	38.3	38	72.9	39.6
4	94.1	51.7	39	151.8	70.0
5	177.6	96.2	40	64.6	32.6
6	104.0	47.3	41	80.0	37.6
7	92.2	36.2	42	102.4	45.0
8	36.0	20.2	43	75.9	34.6
9	181.0	60.1	44	111.1	60.0
10	144.6	56.0	45	96.9	46.3
11	135.5	73.6	46	74.1	43.3
12	172.2	84.7	47	59.2	37.5
13	83.7	33.7	48	61.3	30.8
14	69.5	32.7	49	55.6	23.5
15	175.9	80.6	50	95.7	49.3
16	63.2	29.0	51	97.2	47.3
17	73.8	38.6	52	22.2	9.4
18	298.2	106.9	53	75.2	37.0
19	270.5	116.3	54	65.8	26.6
20	86.5	46.9	55	146.5	69.8
21	143.3	77.7	56	71.6	39.8
22	149.8	76.1	57	34.5	19.6
23	92.0	47.8	58	34.9	14.6
24	99.2	53.7	59	48.2	17.1
25	83.1	47.3	60	48.2	27.5
26	124.0	63.1	61	116.0	59.2
27	65.0	24.1	62	61.8	33.5
28	73.0	30.8	63	63.7	32.5
29	95.6	52.3	64	91.6	51.9
30	48.8	19.4	65	155.0	89.2
31	80.7	33.0	66	50.2	31.0
32	90.8	34.3			
33	43.4	19.8			
34	83.1	37.6			
35	42.7	21.0			
Mean	**114.4**	**52.9**	***Mean***	**80.7**	**40.9**
s.d.	**61.3**	**27.2**	***s.d.***	**33.4**	**17.7**
Min	**36.0**	**19.4**	***Min***	**22.2**	**9.4**
Max	**298.2**	**116.4**	***Max***	**155.0**	**89.2**
